# Long-term outcome of ultrasound-guided focused ultrasound ablation for gestational trophoblastic neoplasia in the cesarean scar: a case report

**DOI:** 10.1186/s12905-022-02114-0

**Published:** 2022-12-15

**Authors:** Dacheng Qu, Yan Chen, Jing Jiang, Qiuling Shi, Honggui Zhou, Zhibiao Wang

**Affiliations:** 1 State Key Laboratory of Ultrasound in Medicine and Engineering, College of Biomedical Engineering, Chongqing Medical University, Chongqing, 400016 People’s Republic of China; 2 Chongqing Key Laboratory of Biomedical Engineering, Chongqing Medical University, Chongqing, 400016 People’s Republic of China; 3 Department of Obstetrics and Gynecology, Affiliated Hospital of North Sichuan Medical College, Nanchong, 637000 People’s Republic of China; 4 Non-invasive and Micro-invasive Laboratory of Gynecology, Affiliated Hospital of North Sichuan Medical College, Nanchong, 637000 People’s Republic of China; 5 School of Public Health and Management, Chongqing Medical University, Chongqing, People’s Republic of China

**Keywords:** Gestational trophoblastic neoplasia, Cesarean scar, High intensity focused ultrasound, Noninvasively, Uterus preservation

## Abstract

**Background:**

The treatment of gestational trophoblastic neoplasia (GTN) is one of the success stories in medical oncology. GTN in the cesarean scar is a rare entity, but most cases need to be treated with hysterectomy or localized uterine lesion resection because of chemoresistant lesions and/or massive bleeding. We present a patient with post-molar GTN in the cesarean scar who was non-invasively treated with ultrasound-guided high intensity focused ultrasound (HIFU) to preserve the uterus and fertility.

**Case presentation:**

A 32-year-old woman was diagnosed with low-risk GTN (FIGO Stage I: 2 prognostic score) after partial hydatidiform mole. The 5th cycle of chemotherapy was interrupted because of persistent hepatic toxicity and impaired ovarian reserve function. However, the uterine lesion persisted (diameter of residual uterine lesion in the cesarean scar: 2.0 cm). Therefore, ultrasound-guided HIFU treatment was performed. A significant gray-scale change was observed during the HIFU treatment. Color Doppler ultrasonography and contrast-enhanced ultrasound (CEUS) was performed to evaluate the ablation effectiveness. Color Doppler ultrasonography showed disappearance of the signal of vascularity and CEUS showed no perfusion in the lesion located in the cesarean scar. The uterine lesion was obviously shrunken one month after HIFU treatment. Menstrual cycle resumed 48 days after HIFU. HIFU treatment decreased the number of chemotherapy cycles and there was complete disappearance of the GTN lesion at 4-month follow-up. The patient has shown no signs of recurrence as of 58-month follow-up.

**Conclusion:**

Ultrasound-guided HIFU may be a useful alternative to lesion resection for GTN in the cesarean scar in patients who show chemoresistance or are not suitable for chemotherapy. It has the potential to ablate the residual uterine lesion noninvasively to preserve the uterus and fertility, avoiding perioperative risks of lesion resection, especially acute bleeding.

## Background

Gestational trophoblastic neoplasia (GTN) is a solid tumor that can be diagnosed without histologic evidence in patients with typical clinical, laboratory, and radiographic features [1]. GTN in the cesarean scar is a special subtype reported only in 45 patients till date [2–9]. Chemotherapy is the primary treatment for GTN in the cesarean scar. However, hysterectomy and localized uterine lesion resection were performed in as many as 71% patients during chemotherapy because of chemoresistant lesions and/or acute bleeding [2].

Due to the similar lesion location and bleeding risk, the successful treatment experiences of cesarean scar pregnancy (CSP) can help inform the treatment strategy for GTN in the cesarean scar. After the pretreatment with uterine artery embolization (UAE) or high intensity focused ultrasound (HIFU), hysteroscopic procedure is a safe and effective procedure for the management of CSP [10, 11]; the reported rates of excessive hemorrhage (> 500 mL) and hysterectomy were 1.66% and 0.28%, respectively. Combined UAE—hysteroscopic diode laser surgery is feasible and safe without anesthesia and cervical dilatation [12].

HIFU is a non-invasive treatment in which the mechanism of therapeutic effect involves thermal and cavitation effects [13]. Many studies have demonstrated the effectiveness and safety of HIFU in the treatment of solid tumors, such as prostate cancer [14], liver tumors [15], recurrent ovary cancer and metastatic pelvic tumors [16], and so on. Available evidence suggests that HIFU can be considered as a fertility-sparing treatment for women with uterine fibroids. These patients were shown to achieve full-term pregnancy with no major perinatal complications or additional obstetric risks [17, 18]. Good pregnancy outcomes can be achieved even in patients with submucous leiomyomas wherein the HIFU ablation energy is in close proximity to the endometrium.

In this case report, we present a patient with GTN in the cesarean scar who was successfully treated with HIFU with preservation of uterus and fertility. In addition, we review the pertinent literature and explore the value of HIFU as a viable fertility-sparing alternative to invasive lesion resection.

## Case presentation

**Fig. 1:**
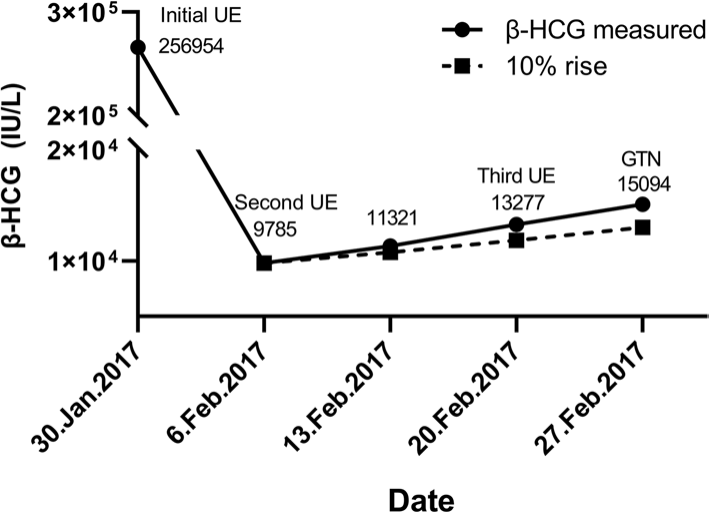
Changes in serum β-hCG level after initial uterine evacuation of partial hydatidiform mole. β-hCG, beta human chorionic gonadotropin; UE, uterine evacuation; GTN, gestational trophoblastic neoplasia

A 32-year-old woman with one previous cesarean section developed post-molar GTN. She complained of cessation of menstruation for 68 days and irregular vaginal bleeding for 10 days. The uterine size was equivalent to 12 weeks gestation, and the beta human chorionic gonadotropin (β-hCG) level was 265,954 IU/L. The patient underwent initial uterine evacuation and the diagnosis of partial hydatidiform mole was confirmed by histopathological examination. Repeat uterine evacuation was performed 1 week later, and the third uterine evacuation was performed 3 weeks later because of the increase in β-hCG level. One month later, she was referred to our hospital as a case of post-molar GTN with β-hCG levels showing an increase of ≥ 10% on each of the 3 successive measurements made over a period of 2 weeks, from 13 to 2017 to 27 Feb 2017 (Fig. 1). The patient had no other symptoms, such as irregular vaginal bleeding, abdominal pain, cough, hemoptysis, or headache. There was no visible lesion in the lower genital tract. The β-hCG level was 15,094 IU/L. Pelvic magnetic resonance imaging (MRI) and transvaginal sonography (TVS) showed the uterine lesion located in the anterior cesarean scar. The size of the uterine lesion was 2.8 cm. The chest CT was normal. A diagnosis of low-risk GTN (FIGO Stage I: 2 prognostic score) was established.

**Fig. 2:**
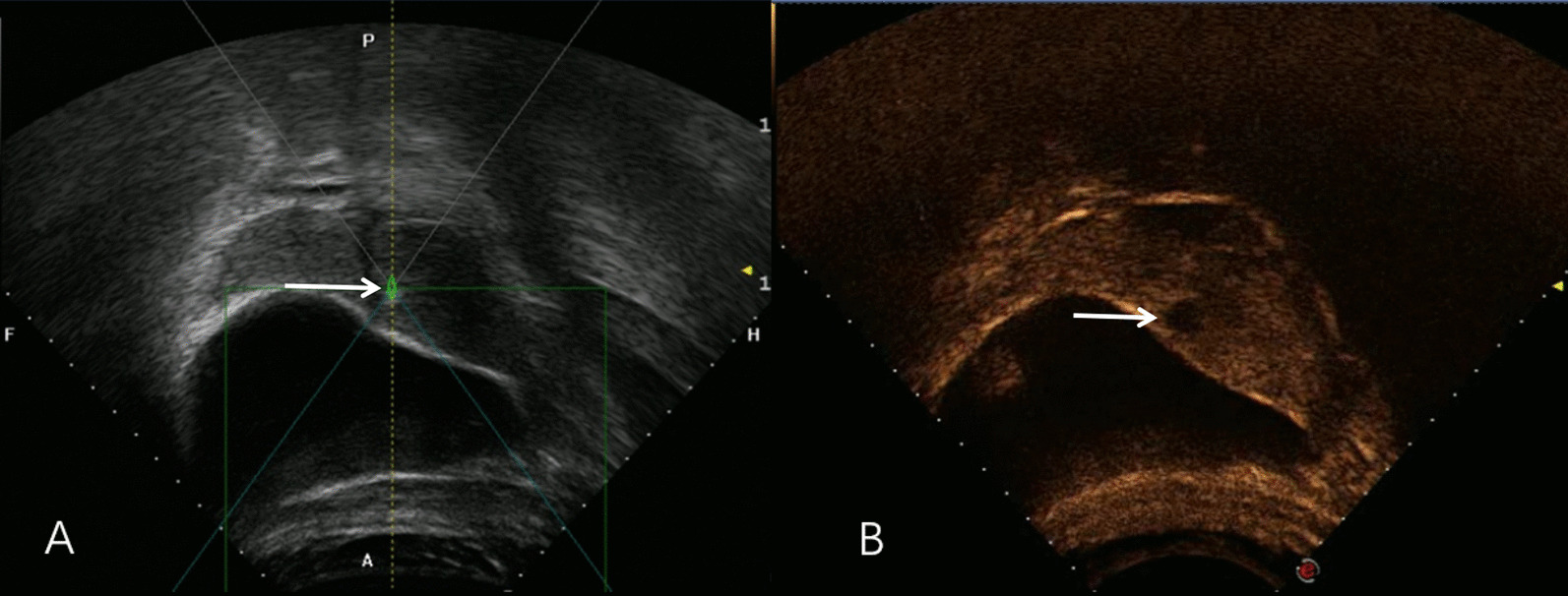
Ultrasound-guided HIFU treatment. Lumpy gray-scale change was seen during the surgery (**A**), while the vascular flow disappeared and the contrast-enhanced ultrasound showed no perfusion of uterine lesion immediately after the surgery (**B**)

**Fig. 3:**
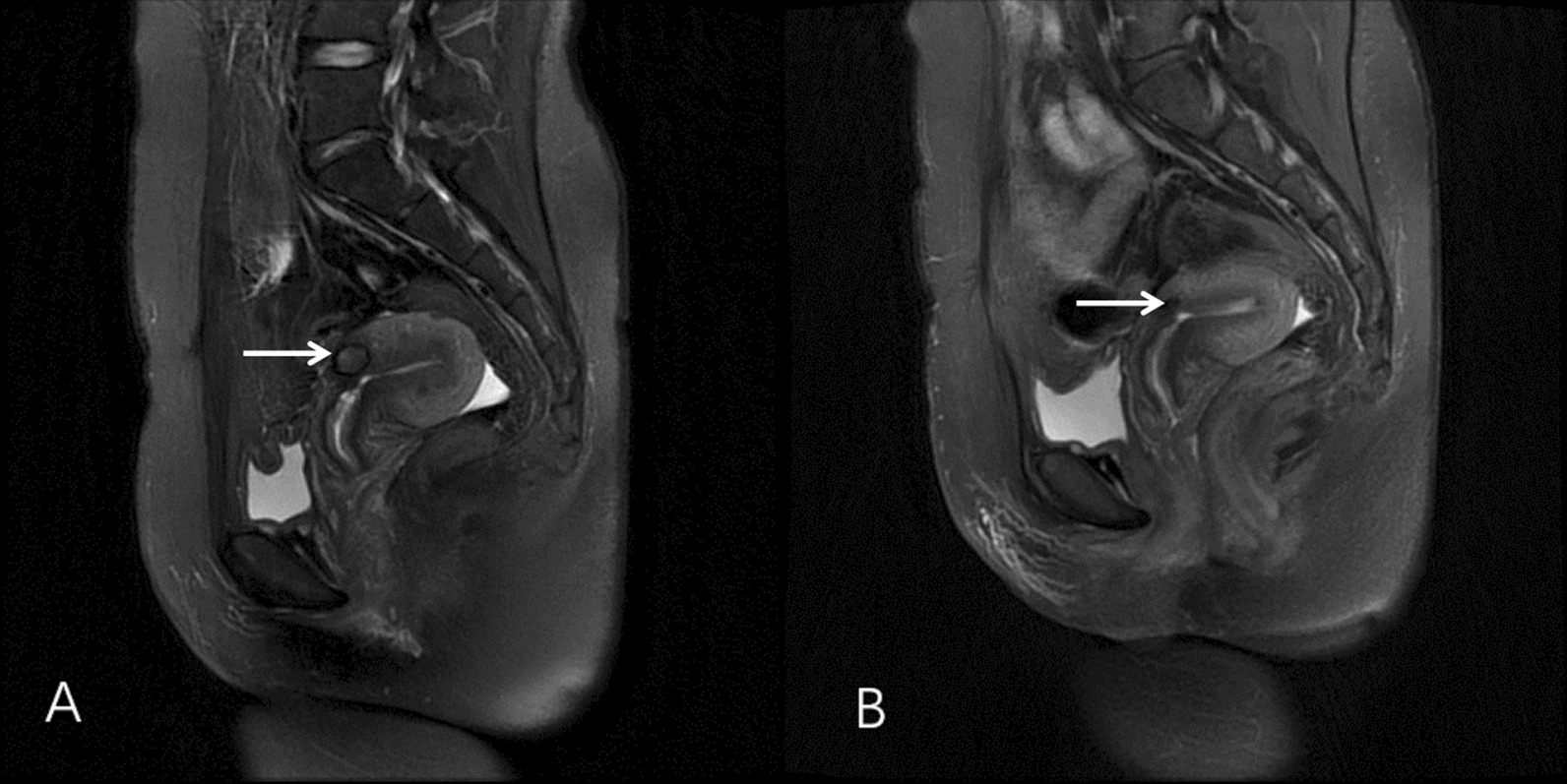
MRI of the uterine lesion pre-HIFU (**A**) and post-HIFU (**B**) treatment. Sagittal T2 image showing the uterine lesion of GTN located in the cesarean scar (**A**). Only cesarean scar defect was seen at 4 months after HIFU treatment (**B**). MRI, magnetic resonance imaging; HIFU, high-intensity focused ultrasound; GTN, gestational trophoblastic neoplasia

We used EP (etoposide, cisplatin) every 3 weeks because the patient was allergic to methotrexate and dactinomycin was not available at our hospital. The hCG returned to normal level after three courses of chemotherapy. The 5th cycle of chemotherapy was interrupted because of persistent hepatic toxicity (Table 1) and damage of ovarian reserve function (Table 2). However, the uterine lesion persisted (size of the residual uterine lesion: 2.0 cm) (Fig. 2A). Therefore, ultrasound-guided HIFU treatment was performed using a Focused Ultrasound Tumor Therapeutic System (Model-JC200, Chongqing Haifu Medical Technology Co. Ltd., Chongqing, China). The patient was positioned prone on the HIFU table, with the anterior abdominal wall in contact with degassed water. A degassed water balloon was placed between the abdominal wall and the transducer to compress and push the bowel away from the acoustic pathway. Point sonication was used, and power was set between 300 and 400 watts. The sonication time was 320 s and the energy delivered was 122,000 J. During the HIFU treatment, a significant gray-scale change was observed. Color Doppler ultrasonography and contrast-enhanced ultrasound (CEUS) was performed to evaluate the effectiveness of ablation. Color Doppler ultrasonography showed disappearance of the vascular signal and CEUS showed no perfusion in the uterine scar lesion (Fig. 3). One month after HIFU treatment, the uterine lesion was found to have reduced to 1.0 cm in diameter. The liver function had returned to normal (Table 1) along with improvement in the ovarian reserve function (Table 2). Her menstrual cycle resumed 48 days after HIFU. At 4-month follow-up after HIFU, the uterine lesion was found to have completely disappeared (Fig. 2B). Hysteroscopy showed absence of lesion in the cesarean scar and no signs of intrauterine adhesion. The patient showed no signs of recurrence as of follow-up conducted at 58 months after HIFU.

**Table 1:** Changes in serum liver enzyme levels in response to chemotherapy

	ALT, U/L	AST, U/L
23 Mar 2017	15	18
13 Apr 2017	60	72
4 May 2017	122	113
25 May 2017	222	181
15 June 2017	202	133
15 July 2017	156	107
20 Aug 2017	90	36
28 Sep 2017	17	19

*ALT* alanine aminotransferase; *AST* aspartate aminotransferase

**Table 2:** FSH and LH levels after chemotherapy and after HIFU treatment

	Post-chemotherapy (pre-HIFU treatment)	Post-HIFU treatment
FSH, mIU/mL	46.45	25.32
LH, mIU/mL	36.06	43.09
E2, pg/mL	95	403

*FSH* follicle stimulating hormone; *LH* luteinizing hormone; *E2* estradiol

## Discussion and conclusions

Correct primary diagnosis is the cornerstone of treatment for GTN in the cesarean scar, and can prevent severe complications of massive bleeding and uterine perforation [2–9]. Abnormal vaginal bleeding, increase in β-hCG level, typical imaging signs on TVS and MRI can facilitate a diagnosis of GTN in the cesarean scar [1, 2]. However, the primary diagnosis of GTN in the cesarean scar may be difficult in some cases. In a study of 31 cases [2], the primary diagnosis was incorrect or unclear in 11 (35%) patients.

In the present study, the patient was asymptomatic. TVS showed a uterine mass with surrounding vascular flow. MRI indicated the size and the location of mass, along with the adjacent areas, especially the cesarean scar defect. MRI is our preferred imaging modality for diagnosis of GTN in the cesarean scar.

Previous cesarean section was shown to be a strong risk factor for occurrence of post-molar GTN and invasive mole [19]. In the present study, two uterine evacuations were conducted before the increase in β-hCG level. We believe that invasion of the tissues around the cesarean scar defect during uterine evacuations may have induced the development of GTN in the cesarean scar.

Chemotherapy is the primary treatment for GTN in the cesarean scar. However, uterine lesion in the cesarean scar area is difficult to be absorbed because of the thin myometrium at this site. Moreover, chemotherapy resistance occurs easily, but the combination of chemotherapy with hysterectomy or localized uterine lesion resection can achieve a good prognosis. According to a study by Wang, complementary hysterectomy and localized uterine lesion resection were performed in 18 (58%) and 4 (13%) patients, respectively, mainly because of chemoresistant lesions [2]. One woman experienced four episodes of relapse and died of tumor progression 39 months after initial laparoscopic uterine lesion resection. Among the 45 reported cases of GTN in the cesarean scar, hysterectomy was performed in 24 (53%) patients and the uterus was preserved in 21 (47%) patients [2–9]. Preservation of the uterus helps preserve fertility, and 2 of 4 women who attempted pregnancy conceived [2]. Due to the thin myometrium in the cesarean scar and extremely abundant vascularization of GTN, localized uterine lesion resection may need to be converted to hysterectomy because of massive intraoperative hemorrhage [3]. In addition to the risk of massive hemorrhage, the possibility of relapse is a key concern while opting for localized uterine lesion resection. In the 21 patients with uterus retention, 8 patients underwent localized uterine lesion resection [2, 3, 8, 9], 1 patient underwent hysteroscopic resection, 1 patient underwent laparoscopic resection, 1 patient underwent transabdominal resection, while the surgical approach was not reported for 5 patients. Another patient was diagnosed as having placental site trophoblastic tumor after hysteroscopic lesion resection and subsequently underwent hysterectomy [3]. In the two cases with hysteroscopic resection, UAE was performed preoperatively due to concerns about bleeding. This may be the reason why patients who underwent local lesion resection did not have massive hemorrhage.

Although there is no clear consensus on the effect of UAE on fertility, UAE may lead to impairment of ovarian reserve and severe intrauterine adhesions [20–22]. UAE is not recommended as the first choice for patients who are desirous of preserving fertility. Several large studies have demonstrated the safety of HIFU in the treatment of benign uterine tumors [23, 24]. Compared with UAE, HIFU does not affect the ovarian function through changes in anti-müllerian hormone (AMH) levels [20, 25].

HIFU has been used as an adjuvant surgical procedure in GTN with chemoresistance or recurrence [26]. A combination of HIFU with chemotherapy was found to be effective for GTN, which can not only reduce the hCG level, but also reduce uterine lesion. However, there are no long-term results. In the present study, chemotherapy had to be discontinued because of drug toxicity. Considering the patient’s expectation of future fertility and minimal trauma to the patient, HIFU was conducted instead of local lesion resection or hysterectomy to treat the residual uterine lesion. The effectiveness of HIFU can be assessed by CEUS immediately after the procedure based on the change in lumpy gray-scale, disappearance of vascular flow, and absence of perfusion of uterine lesion. If the HIFU procedure was ineffective, hysterectomy or localized uterine lesion resection will be selected soon. In our patient, the ovarian reserve function improved and the liver function returned to normal after cessation of chemotherapy. One month after HIFU, the uterine lesion had significantly reduced from 2 cm to 1 cm with a volume reduction to 1/8. The uterine lesion disappeared in 4 months, which was consistent with a previously reported case [26]. The rapid recovery of menstruation and hysteroscopy findings suggest no damage to the endometrium and no intrauterine adhesion. The relatively long-term follow-up of 58 months also confirmed the effectiveness and safety of HIFU. Thus, HIFU can help preserve the uterus and fertility in such patients. To our knowledge, this is the first study to report the treatment of HIFU for GTN in the cesarean scar. In this patient, HIFU not only precluded the need for localized uterine lesion resection, but also helped decrease the courses of chemotherapy.

Ultrasound-guided HIFU may be a viable alternative to lesion resection for GTN in the cesarean scar in patients who show chemoresistance or are not suitable for chemotherapy. It has the potential to ablate the residual uterine lesion noninvasively, preserve the uterus and fertility, and avoid perioperative risks of lesion resection, especially acute bleeding. HIFU helped reduce the courses of chemotherapy and no relapse was observed on long-term follow-up.

## Data Availability

The authors support data transparency.

## Abbreviations

GTN: Gestational trophoblastic neoplasia
HIFU: High intensity focused ultrasound
CEUS: Contrast-enhanced ultrasound
β-hCG: Beta human chorionic gonadotropin
MRI: Magnetic resonance imaging
TVS: Transvaginal sonography
UAE: Uterine artery embolization
AMH: Anti-müllerian hormone
